# Effects of Continuous Glucose Monitoring on Glycemic Control, Mental Health and Self-Management in Adults with Type 1 Diabetes: A Randomized Controlled Trial

**DOI:** 10.3390/healthcare13243197

**Published:** 2025-12-05

**Authors:** Rocío Romero-Castillo, Manuel Pabón-Carrasco, Shakira Kaknani-Uttumchandani, José Antonio Ponce-Blandón

**Affiliations:** 1 Nursing Department, Faculty of Nursing, Physiotherapy and Podiatry, University of Seville, 41009 Seville, Spain; japonce@us.es; 2 Nursing Department, Faculty of Health Sciences, University of Málaga, 29071 Malaga, Spain; shakira@uma.es

**Keywords:** type 1 diabetes mellitus, self-management, continuous glucose monitoring, depression, anxiety

## Abstract

**Highlights:**

**What are the main findings?**

- The *Diabself-care* program, a nurse-led structured diabetes self-management intervention, improved glycemic control, self-care adherence and psychological well-being in adults with type 1 diabetes using continuous glucose monitoring (CGM).
- Higher self-management adherence was a protective factor against both anxiety and depression.

**What are the implications of the main findings?**

- Integrating emotional support and CGM feedback within health promotion produces synergistic effects on both glycemic control and psychological outcomes.
- The results highlight the dual clinical and mental health value of holistic diabetes education.

**Abstract:**

**Background/Objectives**: Adults with type 1 diabetes (T1D) often experience psychological distress that interferes with their ability to maintain optimal self-care. The purpose of this study was to evaluate the effectiveness of the *Diabself-care*, a nurse-led structured diabetes self-management education (DSME) intervention designed to improve glycemic control, self-care practices and mental health among adults with T1D. **Methods**: A total of 224 adults with type 1 diabetes were randomized and final analyses included 110 participants in the intervention group and 106 in the control group. The intervention group received the *Diabself-care* program, consisting of five daily 90 min sessions integrating education, skill training, self-management and coping strategies in addition to usual care. The control group received standard diabetes care. Outcomes were assessed at baseline, 1 month and 3 months. The primary measure was glycemic control and secondary outcomes including self-management, anxiety and depressive symptoms. **Results**: The intervention group achieved a significant increase in time in range at both 1 and 3 months. Self-management adherence improved significantly in the intervention group (*p* < 0.001). Anxiety and depression scores decreased significantly in the intervention group at 1 and 3 months, while they remained unchanged in controls. Regression analyses identified depressive symptoms as the strongest predictor of anxiety (OR = 4.34, 95% CI = 2.99–6.28, *p* < 0.001), while female sex, older age, and low self-management were predictors of depression. Belonging to the intervention group was strongly protective against depression (OR = 0.11, 95% CI = 0.05–0.24, *p* < 0.001). **Conclusions**: The *Diabself-care* program significantly improved glycemic control, self-management, and psychological outcomes in adults with T1D. These findings highlight the dual clinical and mental health benefits of structured nurse-led DSME, supporting its integration into routine diabetes care. The trial is registered at ClinicalTrials.gov, ID: NCT05159843.

## 1. Introduction

Diabetes mellitus is internationally recognized as a public health problem due to its high prevalence, impact on daily life, acute and chronic complications and the resulting healthcare costs. The global prevalence is estimated at 10.5% (537 million people) and is projected to rise to 11.3% by 2030 and 12.2% by 2045 [1]. The diagnosis of diabetes can have a profound effect on quality of life, sometimes leading to social isolation, psychosocial repercussions, uncertainty about the future and concerns regarding potential short- and long-term complications [2]. People with type 1 diabetes (T1D) are at increased risk of mental disorders. The likelihood of depression is twice as high as in the general population, with a prevalence of depressive symptoms of 30%, a prevalence of major depression of 11%, and an anxiety prevalence of 14% compared to 3–4% in the general population [3]. Depression in diabetes is associated with poor treatment adherence, suboptimal glycemic control, increased complications, and higher healthcare costs [4]. Mental disorders are also linked to reduced quality of life, diabetes-related distress, and eating disorders [5].

Managing T1D can be particularly stressful, as it requires a holistic approach encompassing medical, psychological, and social dimensions. Approximately one-third of adults with T1D report high levels of diabetes distress. Acute blood glucose fluctuations, especially hypoglycemic events, can negatively impact mental health, often causing anxiety [6]. Fear of hypoglycemia is common and can impair normal functioning in diabetic patients [7]. In recent years, glycemic control has improved using continuous glucose monitoring (CGM) systems. These devices provide real time glucose readings and alarms for hypo and hyperglycemia, improving safety and effectiveness of therapy, reducing the incidence and duration of hypoglycemia, and decreasing glycemic variability [8]. These improvements contribute to enhanced patient safety, reduced anxiety, and better quality of life [9].

Despite technological advances and improved pharmacological treatments, many patients still exhibit self-care deficits, and personal health beliefs can influence illness perceptions. Education level and disease awareness have been associated with self-care adherence [10]. Therefore, a biomedical model focused solely on pharmacological treatment and the clinical-biological aspects of the disease are insufficient. Instead, it is necessary to incorporate therapeutic education based on the biopsychosocial model [11,12].

Engagement in therapeutic education and diabetes self-management is associated with improved health outcomes, including better glycemic control, reduced complications, lower health risks, and enhanced quality of life. The terms self-management and self-care refer to the activities an individual undertakes to control or reduce the impact of a disease on their health and wellbeing, aiming to prevent complications or comorbidities. In diabetes, self-management includes adherence to a healthy diet, compliance with treatment, regular monitoring, risk reduction, and an active lifestyle [13].

Several studies have demonstrated the effectiveness of Diabetes Self-Management Education and Support (DSMES) in improving clinical outcomes [14]. Type 1 diabetes is a chronic disease that requires a high degree of self-care responsibility, and nurses are well-trained to provide therapeutic education, ongoing monitoring, and personalized feedback [15]. Educators should provide knowledge, foster skills and positive attitudes through dialogue, reasoning, and mutual agreement, offer evidence-based information, and empower patients to take an active role in their treatment [16].

Structured education programs such as DAFNE and T1-REDEEM have shown improvements in glycemic control and emotional well-being in adults with T1D. However, most interventions have focused on either metabolic or psychological outcomes separately, and few have integrated CGM use, self-management and mental health outcomes simultaneously [17,18,19].

Educational interventions for T1D have generally focused on diet, insulin management, CGM use, complication management, and emotional wellbeing. Most have been delivered by nurses in face-to-face group sessions (3–10 participants) lasting 30 to 120 min [20,21,22]. However, despite these initiatives, adherence to recommendations remains low to moderate [23]. More experimental research is warranted to provide high-quality evidence and assess the impact of structured educational programs.

Given the limited evidence on the impact of structured therapeutic education programs that integrate self-management, mental health support, and CGM use in adults with T1D in Spain, this study aimed to evaluate the effect of the *Diabself-care* program on glycemic control (primary endpoint: time in range), self-management, and mental health outcomes. Few studies have simultaneously analyzed glycemic outcomes, self-management behaviours and mental health in adults with T1D using CGM systems. This study addresses this gap by focusing specifically on this population AND providing a comprehensive view of the technological and psychosocial dimensions of diabetes management. The primary objective was to evaluate the effect of the *Diabself-care* program on glycemic control, with time in range (TIR 70–180 mg/dL) at 1 and 3 months as the primary endpoint. Secondary objectives were to assess changes in diabetes self-management and mental health.

## 2. Materials and Methods

### 2.1. Design

This study was a single-blind, parallel-group, two-arm randomized controlled trial (RCT) comparing the effectiveness of a therapeutic education program (*Diabself-care*) versus standard care in adults with T1D using CGM. The primary investigator designed the *Diabself-care* program and provided detailed instructions to the diabetes nurse regarding the session content, scheduling, and teaching methodology.

### 2.2. Setting and Recruitment

The trial was conducted at Virgen del Rocío University Hospital, a regional tertiary-care public hospital that provides comprehensive coverage across all medical specialties of the Spanish National Health System. It is a public hospital belonging to the Andalusian Health Service, located in Seville, in the autonomous community of Andalusia, Spain.

Participants were recruited from the Endocrinology outpatient clinics at the Diagnostic and Treatment Center and from the Diabetes Day Hospital of Virgen del Rocío University Hospital. Inclusion criteria were (a) age > 18 years; (b) diagnosis of T1D; (c) ability to read and speak Spanish; and (d) no significant hearing or visual impairment and no terminal illness. Exclusion criteria were (a) pregnancy; (b) participation in another concurrent interventional study; (c) inability to attend follow-up visits during the study period.

All participants provided written informed consent and were informed of their right to withdraw at any time without consequences. Anonymity and confidentiality were guaranteed in accordance with data protection regulations. The study design and procedure adhered to the Declaration of Helsinki. Informed consent was obtained from all participants prior to the study. Ethical approval was granted by the Biomedical Research Ethics Committee of the Andalusian Regional Government (2231-N-21). The trial is registered at ClinicalTrials.gov, ID: NCT05159843 (https://clinicaltrials.gov (accessed on 3 December 2021)).

### 2.3. Sample Size Calculation, Randomization and Blinding

Sample size calculations were performed in G*Power (version 3.1) for a two-tailed independent-samples t-test comparing mean time in range (TIR 70–180 mg/dL) at the 3-month follow-up between the intervention and control groups. Based on previous diabetes self-management education studies reporting medium effects of TIR, a Cohen’s d = 0.50 was assumed, with α = 0.05, power (1 − β) = 0.80, and an anticipated attrition rate of 20% [24]. Under these parameters, the required total sample was 100 participants (50 per group).

Randomization was performed using a computer-generated allocation sequence in a 1:1 ratio. To ensure allocation concealment, the randomization results were sealed in numbered envelopes and distributed in order of enrollment by another individual who was not involved in the study. The data evaluators and assessors were blinded to the treatment allocation. Due to nature of the intervention, blinding of participants and educators was not feasible. However, outcome assessors remained blinded to group assignments throughout the study to minimize assessment bias.

**Figure 1:**
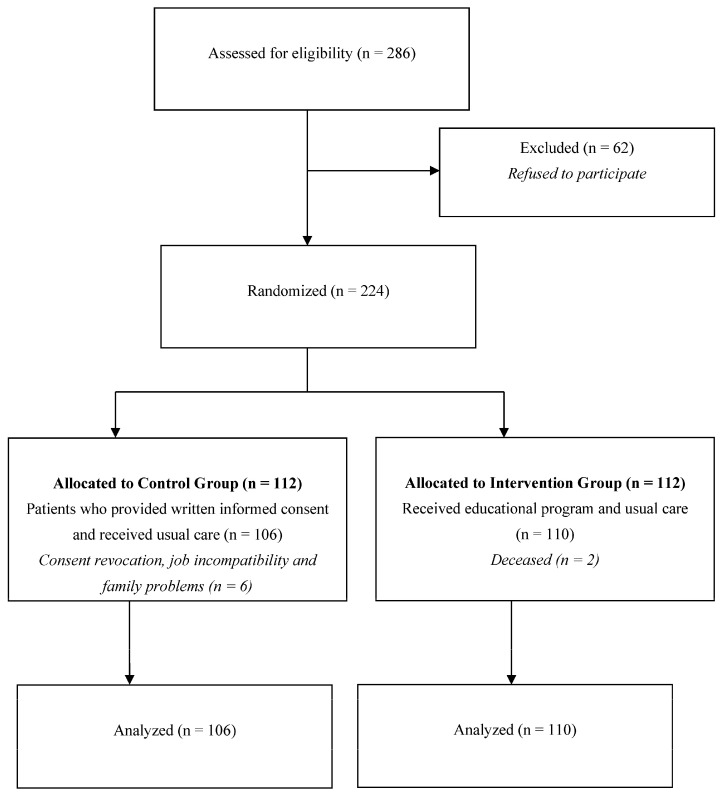
Flow chart of participant selection.

A total of 286 eligible patients were approached and 78.3% (*n* = 224) agreed to participate. These participants were randomized into two groups (intervention and control). In the control group, 106 participants completed the study and 6 withdrew consent for work or family reasons. There were two losses to follow-up in the intervention group and finally, 110 subjects were analyzed in that arm (Figure 1).

### 2.4. Nurse-Led Intervention

The *Diabself-care* program consisted of five consecutive daily sessions of structured therapeutic education, each lasting 90 min, delivered to the intervention group in addition to standard diabetes care. The *Diabself-care* program was theoretically underpinned by the biopsychosocial model, which emphasizes the integration of biomedical, psychological, and social factors in chronic disease management. Each session combined knowledge delivery, skills training, and reflective discussion, and followed a standardized format:

- Didactic component (30 min)—nurse-led presentation of the session topic.
- Practical component (30 min)—simulation and hands-on activities for skill acquisition.
- Discussion and wrap-up (20 min Q&A + 10 min summary)—opportunity for participants to clarify doubts, reflect on their learning, and consolidate key takeaways.

The sessions were delivered by a nurse specialized in diabetes care, trained in the *Diabself-care* protocol to ensure fidelity and consistency of delivery. The primary investigator provided protocol training, oversaw session content, and coordinated the teaching methodology. A trained assistant supported the facilitator in time management, participant engagement and documentation. Periodic review meetings were held, and selected sessions were systematically reviewed through recorded session evaluations to ensure consistency, adherence to the protocol and quality of delivery throughout the intervention.

Educational content, summarized in Supplementary Material S1, covered essential aspects of diabetes self-management, including insulin adjustment, dietary planning, CGM interpretation and management, hypoglycemia prevention and treatment, emotional wellbeing, and strategies for integrating self-care into daily life.

### 2.5. Current Practice (Usual Care)

All participants report to the Diabetes Day Hospital for their scheduled clinic appointments with their diabetes doctors and nurses’ educators, which is the current standard care throughout the study period. Standard care included routine clinical follow-up, review of CGM data, and general counseling on diabetes self-management according to clinical practice guidelines. The control group did not receive any additional or structured educational intervention beyond this usual care during the study period.

### 2.6. Data Collection

Data were collected at baseline, one month and three months after intervention by two volunteer registered nurses who were blinded to participants’ group allocations. Collected data included CGM metrics, clinical measurements and self-reported questionnaires.

### 2.7. Measurements and Instruments

Demographic characteristics were collected, including age, sex, employment status, marital status and education level.

Glycemic control was assessed using the CGM data. The parameters recorded included: percentage of time above range (>180 mg/dL), time in target range (70–180 mg/dL), time below range (<70 mg/dL), average glucose (mg/dL) and glycated hemoglobin (HbA1c, %).

Self-reported questionnaires were administered to collect demographic characteristics, assess anxiety and depressive symptoms, and evaluate adherence to self-management practices. Anxiety and depressive symptoms were measured using the Goldberg Anxiety and Depression Scale, which comprises two subscales of nine dichotomous (yes/no) items each. The cut-off score for the anxiety subscale is ≥4, and for the depression subscale is ≥2, with higher scores indicating greater symptom severity. In previous research, the anxiety subscale demonstrated a Cronbach’s alpha of 0.75, and the depression subscale a Cronbach’s alpha of 0.73. Reliability coefficients were 0.73 and 0.78, respectively, both within the acceptable range. The full scale shows 91% specificity and 86% sensibility [25].

Self-management was measured using the Summary of Diabetes Self-Care Activities (SDSCA) questionnaire [26], which assesses the frequency of self-care behaviors over the previous seven days. The Spanish version of the instrument is culturally appropriate, valid and reliable among Spanish patients with diabetes (Cronbach’s alpha = 0.62) [27]. In this study, including only the domains of diet, physical activity and blood glucose monitoring, as these were the behaviors directly targeted by the educational intervention. This version consists of 7 items, assessing the frequency of each self-care behavior over the previous seven days. Responses range from 0 to 7 days, and the total score ranges from 0 to 49 points, with higher scores indicating better adherence to diabetes self-care behaviors. The SDSCA does not use predefined cut-off points; instead, results are interpreted in relation to baseline values and between-group comparisons.

### 2.8. Statistical Analyses

Statistical analyses were conducted using SPSS version 29 (SPSS Inc., Chicago, IL, USA) software program. Continuous variables are expressed as mean (SD) and categorical variables as number and percentage. Between-group comparisons of baseline sociodemographic and clinical characteristics were performed using independent-samples t tests for continuous variables and chi-square tests for categorical variables. A *p*-value ≤ 0.05 was considered statistically significant.

To evaluate longitudinal changes in the primary and secondary outcomes, we used two-way repeated-measures ANOVA, with time (baseline, 1 month, and 3 months) as the within-subject factor and study group (intervention vs. control) as the between-subject factor. Mauchly’s test of sphericity was examined, and when the assumption was violated, Greenhouse-Geisser corrections were applied. We report main effects of time and group, as well as the time × group interaction. When a significant interaction effect was found, planned post hoc pairwise comparisons were conducted to explore within-group changes over time and between-group differences at each time point. These comparisons were performed using paired or independent-samples *t*-tests with Bonferroni-adjusted significance levels.

For exploratory analyses, binary logistic regression was used to identify predictors of anxiety and depression at 3 months. The dependent variables were defined using Goldberg cut-offs (anxiety ≥ 4; depression ≥ 2). Predictors included sex, age, study group, CGM parameters, depressive symptoms (for anxiety model), anxiety symptoms (for depression model), and SDSCA total score. Model fit was assessed using the Hosmer–Lemeshow test, explanatory power with Cox & Snell and Nagelkerke R^2^, and predictive accuracy by percentage correctly classified. Odds ratios (OR) with 95% confidence intervals are reported. All analyses were conducted on the complete dataset. A per-protocol analysis was performed to confirm the robustness of the results.

## 3. Results

As shown in Figure 1, a total of 224 patients agreed to participate in the study and were randomly assigned to either the intervention or control group. In the control arm, 106 participants completed the study, while six withdrew consent due to work or family commitments. In the intervention arm, two participants were lost to follow-up, resulting in a final analysis of 110 individuals in that group.

### 3.1. Baseline Characteristics

Baseline sociodemographic are presented in Table 1. The two groups were comparable at baseline, with no statistically significant differences in age, sex distribution, educational level, marital status, or employment status (*p* > 0.05). Baseline clinical measurements were also similar between groups, with no significant differences in CGM parameters, Goldberg Anxiety and Depression scores, or SDSCA scores, as shown in Table 2.

### 3.2. Primary Outcome—Time in Range

For the primary outcome, time in range (TIR), the two-way repeated-measures ANOVA showed a significant main effect of time (F(2, 420) = 184.32, *p* < 0.001) and a significant time × group interaction (F(2, 420) = 110.00, *p* < 0.001). Post hoc comparisons indicated that TIR increased significantly from baseline to 1 and 3 months in the intervention group, while it remained stable in the control group. At both 1 and 3 months, TIR was significantly higher in the intervention group than in the control group (Bonferroni-adjusted *p* < 0.001) (Table 2).

### 3.3. Secondary Outcomes—Self-Management and Mental Health

The ANOVA revealed improvements in self-care adherence over time (F(2, 420) = 261.98, *p* < 0.001) and a significant group effect (F(1, 210) = 123.72, *p* < 0.001). The time × group interaction was also significant (F(2, 420) = 86.76, *p* < 0.001), showing that self-care behaviors improved more in the intervention group compared with the control group.

For Goldberg anxiety, there were significant effects of time (F(2, 418) = 33.43, *p* < 0.001) and group (F(1, 209) = 234.41, *p* < 0.001), and a highly significant time × group interaction (F(2, 418) = 205.13, *p* < 0.001). Participants in the intervention group showed reductions in anxiety symptoms from baseline to 1 and 3 months, whereas the control group remained largely stable.

For Goldberg depression, both the time effect (F(2, 418) = 113.68, *p* < 0.001) and the group effect (F(1, 209) = 221.25, *p* < 0.001) were significant. However, the time × group interaction was not significant (F(2, 418) = 0.85, *p* = 0.43), indicating that although depressive symptoms improved over time, the rate of change did not differ significantly between groups.

### 3.4. Binary Logistic Regression Analyses

Table 3 presents the model for anxiety at 3 months. Depressive symptoms and SDSCA total score were significant predictors of anxiety, with the model correctly classifying 92% of participants (Nagelkerke R^2^ = 0.831). Higher depressive symptom scores were strongly associated with greater odds of anxiety (OR = 4.34, 95% CI = 2.99–6.28, *p* < 0.001), indicating that each unit increase in depressive symptoms more than quadrupled the likelihood of experiencing anxiety. In contrast, higher self-care adherence (SDSCA total score) was associated with lower odds of anxiety (OR = 0.88, 95% CI = 0.84–0.93, *p* < 0.001), suggesting a protective effect.

Table 4 shows the model for depression at 3 months. Significant predictors included sex, age, study group, and SDSCA total score. Female sex (OR = 2.82, 95% CI = 1.42–5.59, *p* = 0.003) and older age (OR = 1.04 per year, 95% CI = 1.01–1.07, *p* = 0.016) were associated with higher odds of depression. Belonging to the intervention group was strongly associated with lower odds of depression (OR = 0.11, 95% CI = 0.05–0.24, *p* < 0.001). Like the anxiety model, higher SDSCA total scores were protective against depression (OR = 0.88, 95% CI = 0.83–0.92, *p* < 0.001). This model achieved good explanatory power (Nagelkerke R^2^ = 0.528) and correctly classified 80% of participants.

## 4. Discussion

This randomized controlled trial demonstrated that the *Diabself-care* program significant improvements in glycemic control, self-management, and mental health outcomes in adults with T1D. The two-way repeated measures ANOVA showed robust main effects of time and group, together with strong time × group interactions. The magnitude and consistency of the interaction effects confirm that the intervention not only accelerated the rate of improvement but also altered the trajectory of glycemic, behavioral, and psychological outcomes in a way that usual care did not achieve.

Increases in TIR are particularly meaningful from a clinical perspective. Previous studies have shown that every 5% increase in TIR is associated with significant reductions in the risk of microvascular complications, including retinopathy and nephropathy [28,29,30]. Thus, the improvement reported here may have long-term implications for reducing diabetes-related complications. Importantly, our results extend previous evidence by showing that a structured, nurse-led intervention can sustain these benefits over several months, while also alleviating mental health burden, a frequently overlooked dimension of diabetes care.

Our findings are consistent with prior research demonstrating the value DSME programs, particularly those delivered in structured, interactive formats [31]. Trials incorporating education and behavioral support have reported improvements in self-care behaviors and glycemic control [32,33]. However, few interventions have integrated nutrition and exercise education, lifestyle habits, CGM management and mental health within a single structured program. This multimodal approach may explain the robust effects observed on both glycemic and psychological outcomes in the current study.

The use of case-based learning provided participants with experiential opportunities to practice insulin management, dietary planning, and problem-solving. The integration of CGM data allowed participants to visualize and understand the impact of their daily decisions on glycemic variability, reinforcing behavioral changes. This aligns with recent evidence demonstrating that real-time feedback from CGM use not only improves glycemic outcomes but also increases engagement in self-management practices [34].

In addition, dedicated sessions on mental health and stress management promoted coping strategies, which may have mediated the reduction in depressive and anxiety symptoms. Previous trials have highlighted the benefit of incorporating psychological support within DSME programs, reporting reductions in diabetes-related distress and improvements in quality of life [35,36].

The strong predictive role of self-management adherence in regression models supports the hypothesis that improvements in daily self-management behaviors are key drivers of both metabolic and psychological outcomes. This result supports prior evidence showing that improvements observed in structured DSME interventions are largely attributable to enhanced self-care behaviors rather than to medication adjustments [14].

The regression analyses provided additional insight. For anxiety, depressive symptoms emerged as the strongest predictor, confirming the well-documented comorbidity between both conditions in T1D [37]. Higher adherence to self-management behaviors acted as a protective factor, reinforcing the central role of behavioral engagement in emotional well-being. For depression, significant predictors included female sex, older age, and lower adherence to self-management, in line with previous evidence of greater vulnerability among women and older adults with diabetes [38,39]. Importantly, assignment to the intervention group substantially reduced the odds of depression, underscoring the preventive value of structured DSME programs. Taken together, these findings suggest that beyond improving metabolic outcomes, DSME interventions may exert their beneficial effects on mental health.

The association between depressive symptoms, anxiety and self-management behaviors highlights the need for an integrated approach to diabetes care that simultaneously addresses metabolic control and psychological well-being. Routine screening for depressive and anxiety symptoms in adults with T1D could contribute to early identification of vulnerable patients and promote more personalized and comprehensive care strategies. Future research should further explore the longitudinal mechanisms underlying these associations and assess whether improvements in self-management mediate long-term mental health benefits.

In relation to the psychological dimension, Ebert et al. [40] evaluated the 6-month effectiveness of an Internet-based guided self-help program for adults with T1D presenting elevated depressive symptoms. The authors reported significant reductions in depressive symptoms and diabetes-related emotional distress for six months. However, their study did not incorporate objective glycemic monitoring through continuous glucose sensors. In this context, our study extends previous evidence by integrating psychological assessment with self-management evaluation and CGM data. Although several studies have evaluated the long-term effects of structured diabetes education programs over a 12-month follow-up, most of this evidence has been generated in populations with type 2 diabetes [41].

Strengths of this study include the randomized controlled design, the use of CGM-derived outcomes, the inclusion of validated psychological scales and the high completion rate among participants. Nevertheless, some limitations should be acknowledged. The trial was conducted in a single center, which may limit generalizability. Blinding of participants and educators was not feasible due to the nature of the intervention. Finally, self-reported measures may be subject to recall or social desirability bias, although this was mitigated by more objective measurements such as CGM data.

Previous evidence from randomized controlled trials on diabetes self-management education has been largely concentrated on type 2 diabetes, whereas studies in T1D remain scarce. In fact, few trials have explored structured educational programs in this population, despite the central role of self-care in optimizing outcomes. Our own pilot randomized trial previously demonstrated that a structured DSME program promoted significant improvements in glucose levels and self-care behaviors among patients with T1D [9]. This highlights the novelty and relevance of the present study, as it contributes to addressing a well-recognized gap in the literature and reinforces the need for further multicenter RCTs to confirm and expand these findings.

This study contributed novel evidence by focusing specifically on adults with T1D using CGM systems and integrating glycemic control, self-management behaviors, and mental health in a single analytical framework. This integrated perspective is still underrepresented in current literature and offers valuable insights for both clinical practice and the development of digital health interventions.

Given the promising results of structured DSME programs, future research should explore the integration of eHealth tools, such as chatbots or mobile-based self-management support, to enhance scalability, personalization, and long-term sustainability of diabetes care. Multicenter trials with extended follow-up are needed to assess whether improvements in TIR and psychological well-being are maintained over time and whether they translate into reductions in diabetes complications and health care utilization.

## 5. Conclusions

To conclude, this RCT provides novel and robust evidence that *Diabself-care*, a structured nurse-led DSME program integrating health education and CGM feedback can produce clinically meaningful improvements in both glycemic control and mental health in adults with T1D. By demonstrating sustained gains in TIR, enhanced self-management adherence, and reduced anxiety and depressive symptoms, this study highlights the dual clinical and psychological value of comprehensive DSME. These findings make an important contribution to the literature and support the incorporation of structured, holistic education programs into diabetes care. Scaling such interventions through digital health tools may represent the next step toward more accessible, personalized and sustainable diabetes management. Beyond research implications, these results have direct relevance for clinical practice and health policy, underscoring the need to embed structured education into standard care pathways and to provide training for healthcare professionals.

## Figures and Tables

**Table 1:** Baseline sociodemographic characteristics of participants (*n* = 216).

Characteristic	Intervention Group (*n* = 110)	Control Group (*n* = 106)	*X*^2^/*t*	*p*-Value
Age (years), M (SD)	35.68 (11.05)	35.49 (11.23)	0.324	0.698
(Minimum–maximum)	(26–58)	(25–56)		
Sex, *n* (%)			4.185	0.728
Female	57 (51.81)	55 (51.88)		
Male	53 (48.19)	51 (48.12)		
Education, *n* (%)			2.267	0.526
Primary school	49 (44.55)	46 (43.39)		
Secondary school	48 (43.63)	45 (42.45)		
High school	9 (8.18)	10 (9.43)		
University	4 (3.64)	5 (4.73)		
Marital status, *n* (%)			2.312	0.678
Single	43 (39.09)	40 (37.73)		
Married	51 (46.36)	52 (49.05)		
Divorced or widowed	16 (14.55)	14 (13.22)		
Current employment *n* (%)			3.237	0.224
No	48 (43.63)	47 (44.34)		
Yes	62 (56.37)	59 (55.66)		

Abbreviations: M, mean; SD, standard deviation.

**Table 2:** Longitudinal analysis of outcomes using two-way repeated-measures ANOVA (time × group interaction).

Outcome	F_Time (df)	*p*_Time	F_Group (df)	*p*_Group	F_Time × Group (df)	*p*_Time × Group
Target range (%)	184.32 (2, 420)	<0.001	30.25 (1, 210)	<0.001	110.00 (2, 420)	<0.001
High range (%)	278.28 (2, 420)	<0.001	3.14 (1, 210)	0.078	37.67 (2, 420)	<0.001
Low range (%)	38.29 (2, 420)	<0.001	35.70 (1, 210)	<0.001	3.23 (2, 420)	0.041
Goldberg Scale—Anxiety (0–9)	33.43 (2, 418)	<0.001	234.41 (1, 209)	<0.001	205.13 (2, 418)	<0.001
Goldberg Scale—Depression (0–9)	113.68 (2, 418)	<0.001	221.25 (1, 209)	<0.001	0.85 (2, 418)	0.43
SDSCA (0–49)	261.98 (2, 420)	<0.001	123.72 (1, 210)	<0.001	86.76 (2, 420)	<0.001

**Table 3:** Binary logistic regression analysis for anxiety.

Variables	B	SE	Wald	*p*-Value	OR	95% CI for OR
Sex	0.602	0.375	2.571	0.109	1.826	0.875–3.811
Age	0.025	0.017	2.255	0.133	1.026	0.992–1.060
Study group	−0.220	0.473	0.216	0.642	0.803	0.318–2.027
Target range	0.081	0.148	0.300	0.584	1.085	0.811–1.450
High range	0.023	0.149	0.023	0.879	1.023	0.763–1.371
Low range	−0.004	0.164	0.001	0.980	0.996	0.722–1.374
Goldberg depression score	1.467	0.189	60.328	0.000 *	4.335	2.994–6.277
SDSCA total score	−0.127	0.026	24.594	0.000 *	0.881	0.838–0.926
Constant	−2.441	15.882	0.024	0.878	0.087	-
Model summary: Percentage correctly classified = 92%						
Cox & Snell R^2^ = 0.619; Nagelkerke R^2^ = 0.831						
Hosmer-Lemeshow test = 0.060						
Omnibus Test of model coefficients: *p* < 0.001						

Abbreviations: B, beta coefficient, SE, standard error; OR, odds ratio; CI, confidence interval; SDSCA, Summary of Diabetes Self-Care Activities. * Statistically significant.

**Table 4:** Binary logistic regression analysis for depression.

Variables	B	SE	Wald	*p*-Value	OR	95% CI for OR
Sex	1.037	0.349	8.836	0.003 *	2.822	1.424–5.593
Age	0.038	0.016	5.762	0.016 *	1.038	1.007–1.071
Study group	−2.207	0.396	31.031	0.000 *	0.110	0.051–0.239
Target range	−0.135	0.211	0.412	0.521	0.874	0.578–1.320
High range	−0.072	0.212	0.115	0.735	0.931	0.615–1.409
Low range	−0.301	0.217	1.922	0.166	0.740	0.483–1.133
Goldberg anxiety score	−0.048	0.087	0.298	0.585	0.953	0.803–1.132
SDSCA total score	−0.133	0.026	25.480	0.000 *	0.876	0.832–0.922
Constant	19.591	21.760	0.811	0.368	3.224	
Model summary: Percentage correctly classified = 80%						
Cox & Snell R^2^ = 0.385; Nagelkerke R^2^ = 0.528						
Hosmer-Lemeshow test = 0.084						
Omnibus Test of model coefficients: *p* < 0.001						

Abbreviations: B, beta coefficient, SE, standard error; OR, odds ratio; CI, confidence interval; SDSCA, Summary of Diabetes Self-Care Activities. * Statistically significant.

## Data Availability

Due to privacy and ethics committee requirements, the data are not published. Any researcher wishing to access them may contact the corresponding authors.

## Supplementary Materials

The following supporting information can be downloaded at https://www.mdpi.com/article/10.3390/healthcare13243197/s1, Supplementary S1. Sessions and key activities of the *Diabself-care* program.

## Abbreviations

The following abbreviations are used in this manuscript:

CGM	Continuous Glucose Monitoring
DSMES	Diabetes Self-Management Education and Support
RCT	Randomized Controlled Trial
SDSCA	Summary of Diabetes Self-Care Activities
