# Supplementary material for: Effects of Continuous Glucose Monitoring on Glycemic Control, Mental Health and Self-Management in Adults with Type 1 Diabetes: A Randomized Controlled Trial
Source: Healthcare (Basel). 2025 Dec 5;13(24):3197. doi: 10.3390/healthcare13243197 (PMC12732679; doi:10.3390/healthcare13243197)
Supplement: Supplementary file 1 [file healthcare-13-03197-s001.zip › healthcare-3999944-supplementary.pdf]

Supplementary S1. Sessions and key activities of the *Diabself-care* program

Session & contents	Key activities
Session one: Insulin administration and glycemic control<br>• Insulin preparation and administration<br>• Prevention of side effects<br>• Correct and efficient use of CGM sensor<br>• Setting hypo/hyperglycemia alarms	1. Nurse-led education and simulation<br>2. Patient demonstration<br>3. Q&A session<br>4. Summary and key points
Session two: Acute complications management<br>• Metabolic control goals<br>• Hypoglycemia prevention and resolution<br>• Hyperglycemia prevention and resolution<br>• Complications from poor glycemic control	1. Nurse-led presentation<br>2. Practical case-based exercises<br>3. Q&A session<br>4. Summary and key points
Session three: Nutrition<br>• Label reading<br>• Recognition and calculation of carbohydrates<br>• Planning a healthy daily diet<br>• Calculating carbohydrate portions per meal	1. Nurse-led presentation<br>2. Practical exercise: diet planning and carbohydrate counting<br>3. Q&A session<br>4. Summary and key points
Session four: Physical exercise<br>• Physical and psychological benefits<br>• Recommended exercise types<br>• Safety precautions<br>• Exercise and social relationships	1. Nurse-led presentation<br>2. Clinical case discussions<br>3. Guided physical activity<br>4. Q&A session<br>5. Summary and key points
Session five: Mental health self-management<br>• Mental health and diabetes<br>• Relationship with glycemic control<br>• Stress relief strategies<br>• Support networks and resources	1. Nurse-led presentation<br>2. Ice-breaker and relaxation activity<br>3. Experience sharing<br>4. Q&A session<br>5. Summary and key points
